# High Grade Anal Dysplasia in People Living with HIV: A Review of the Anal Cancer/HSIL Outcomes Research (ANCHOR) Trial. Implications for Screening and Treatment Strategies to Minimize Anal Cancer in a Very High-risk Population

**DOI:** 10.53876/001c.77452

**Published:** 2023-06-05

**Authors:** David M. Aboulafia

**Affiliations:** 1 Floyd & Delores Jones Cancer Institute, Virginia Mason Medical Center, Seattle, WA, USA; 2 Division of Hematology, University of Washington, Seattle, WA, USA

**Keywords:** High-grade anal intraepithelial neoplasia, Anal cancer, HIV/AIDS, ANCHOR Study

## Abstract

The rates of AIDS-defining cancers have plummeted for people living with HIV (PLWH) and who have access to highly active antiretroviral therapies. In contrast, as survival of PLWH has improved and now rivals that of age-matched controls, rates of non-AIDS-defining cancers are increasing. Exposure to oncogenic viruses including human papillomavirus (HPV) as well as to traditional carcinogens, such as tobacco and alcohol are among the reasons for many of these cancers. Worldwide, anal cancer rates are increasing, and this is particularly true for high-grade squamous intraepithelial lesions (HSIL) evolving into invasive anal cancer in PLWH. Herein, I briefly review the oncogenic viruses most important in the pathogenesis of AIDS-defining and non-AIDS-defining malignancies and then focus on the link between HPV and anal cancer and efforts to minimize the risk of anal cancer in PLWH. The Anal Cancer/HSIL Outcomes Research (ANCHOR) study is a randomized phase III clinical trial which enrolled nearly 4500 participants across 25 diverse cities in the United States. PLWH who at time of enrollment were 35 years of age or older and who had biopsy-proven HSIL were eligible to take part in the study. The study is the first to show that screening and treating HSIL in a group at high risk for anal cancer can lead to a reduction in anal cancer incidence. Lessons learned from the ANCHOR study may also provide a blueprint for best practices when reaching out and recruiting marginalized groups with cancer into clinical trials. Much work is needed to plan for screening and treatment programs, including better algorithms for referral for high resolution anoscopy (HRA), and increased training to develop a workforce proficient in HRA screening and treatment of anal cancer. The development of progression biomarkers to identify those with HSIL most in need of treatment is essential. Finally, a more detailed analysis of costs and benefits of screen and treat algorithms for this malignancy is necessary for anal cancer screening to be implemented on a global scale.

## INTRODUCTION

The changes in the care of people living with HIV (PLWH) over the past three and a half decades have been transformative. Early in the AIDS epidemic, antiretroviral treatments (ART) were associated with significant problems. Treatment-related side effects were common, the regimens were cumbersome, and there was fear among those affected by HIV that the drugs might hasten death rather than alleviate pain and suffering.^1^ In addition, the duration of benefit of ART was limited to somewhere between 12 and 18 months.^2^ Invariably, with single agent ART, resistance to treatment occurred rapidly. Consequently, it was challenging for PLWH to take their medications as recommended. Patients often succumbed from opportunistic infections, wasting illness, and complications of malignancy.

During these early years of the HIV epidemic, hematologists and oncologists were in a unique position to care for these patients. Their experience with managing illness in people with incurable malignancies and the infectious complications that their chemotherapy regimens provoked provided them with somewhat analogous models of care.^3^ HIV primary care providers, infectious disease specialists, and multi-specialty HIV clinics also shared in the general and subspecialty care of these patients.

A new era in the treatment of PLWH began in late 1995 following the discovery that ART combinations could reliably suppress HIV replication and over time promote immune reconstitution.^4^ The International Conference on AIDS, held in the summer of 1996 in Vancouver, Canada, detailed a series of promising clinical reports of newer combinations of highly active antiretroviral therapies (HAART) that inhibited HIV replication, and which provided dramatic clinical benefits. Suddenly patients who were at the brink of death improved and this seemingly miraculous phenomenon of return to health was dubbed by the lay press and the medical community as the *Lazarus effect*.^5^ The origins of this term can be traced to the biblical story wherein Jesus is said to have raised Lazarus from the dead. With HAART, so too were PLWH getting a second opportunity for life. For the first time in 15 years, there was genuine optimism that physicians and public health officials might have viable tools to combat what had mushroomed into a rapidly growing global pandemic.

Increasingly sensitive assays to accurately quantitate one measure of the immune system (T-cell lymphocyte subsets) and to measure the burden of HIV viral replication from a small quantity of blood (HIV viral load) were also meaningful laboratory innovations that profoundly impacted patient care.^6^ It meant that clinicians for the first time had at their disposal readily available laboratory tests to gauge how their patients were responding to treatments. With these tests, health care workers could better estimate the risk of opportunistic infections and malignancies in this heavily immunosuppressed patient population.

The ability to assess for HIV-1 genetic mutations and replication fitness (HIV genotype and phenotype assays) allowed clinicians further opportunities to customize HAART, particularly as new agents and classes of antiviral therapies were being developed. With resultant immune reconstitution, the incidence of infectious deaths among PLWH declined. As the AIDS epidemic moved into the “HAART era” AIDS-defining and non-AIDS-defining malignancies remained a principal cause of mortality for PLWH.^7–9^

## ONCOGENIC VIRUSES

Many of the cancers diagnosed in PLWH are associated with oncogenic viruses (Table 1).^10,11^ These viruses include Human Herpes Virus Type-8 (HHV-8) which is etiologically linked to Kaposi’s sarcoma as well as several very rare non-Hodgkin’s lymphoma (NHL) variants, and Epstein Barr Virus (EBV) which is associated with roughly a third of AIDS-defining NHLs.^12,13^ EBV is also linked very rarely with leiomyosarcoma.^14^ Both Hepatitis B Virus and Hepatitis C Virus are associated with liver cancer and very rarely with indolent B-cell NHL.^15,16^

Another important oncogenic virus linked to malignancy in PLWH is human papillomavirus (HPV). A double-stranded, non-enveloped DNA virus, HPV invades epithelial tissue. In symptomatic patients, HPV can present with pruritis, tenderness, warts, and precancerous lesions. But in most people, it is a surreptitious and largely asymptomatic infection.^17–19^ Little wonder that HPV is the most common sexually transmitted disease (STD) in the United States among 15- to 59-year-olds.^20^ HPV contributes to the pathogenesis of some head and neck cancers as well as cancers of the anogenital (cervical, vaginal, vulvar, penile, anal) region. The incidence of these cancers in the United States is shown in Table 2.^21^ Anal neoplasia caused by HPV can manifest as preinvasive (squamous intraepithelial lesions [SIL]) or invasive (anal cancer) disease.

## HPV BIOLOGY

HPV DNA contains coding regions for oncogenic E6 and E7 proteins. By inactivating the tumor suppressor protein p53, E6 protein disturbs DNA repair mechanisms and leads to the accumulation of mutations in host cells.^22^ Retinoblastoma protein (pRb) is inhibited by E7 and leads to host cell proliferation.^23^ HPV-associated E6 and E7 proteins are a leading cause of dysplastic changes leading to anal SIL. This risk is amplified in a milieu of co-occurring immuno-suppression which promotes the persistence of HPV infection.

An example of primary immunodeficiency contributing to HPV infection and malignancy is a rare congenital autosomal dominant disorder characterized by the abnormal retention of mature neutrophils in the marrow that results in chronic neutropenia: the WHIM syndrome (Warts, Hypogammaglobulinemia, Infections, and Myelokathexis). Individuals with WHIM syndrome may also have hypogammaglobulinemia and are uniquely susceptible to potentially life-threatening bacterial infections and problematic HPV infections.^24^ Far more commonly, secondary immunodeficiency disorders including HIV, malignancy, severe and chronic malnutrition, and the immunosuppressive medications used to minimize the risk of rejection of transplanted organs may predispose patients to HPV infections and squamous cancers in affected tissues.

The interactions between HIV and HPV are unique and important to appreciate. HIV coinfection promotes HPV-associated squamous malignancies at the molecular level. In laboratory studies, the HIV-encoded Tat protein enhances expression of the HPV E6 and E7 proteins.^25,26^ Furthermore, HIV infection as well as other STDs such as Chlamydia, Gonorrhea, and Herpes Simplex viruses may compromise the genital mucosal epithelial barrier. This can lead to the diffusion of HPV virions through the epithelium and initiate infection through invasion into basal epithelial cells. HPV can further compromise tissue integrity leading to easier passage of HIV virions. In addition, the immune cells activated by HPV infection are also highly vulnerable to HIV infection. When caring for a patient with severe and problematic HPV infection clinicians should bear in mind the possibility of underlying immune dysfunction.^19^

Lesions resulting from mucosal involvement with HPV are classified as either low-risk or high-risk based on risk of transformation to invasive cancer. Low-grade SIL (LSIL) is present clinically as warts and is most frequently associated with HPV 6 and 11. High-grade SIL (HSIL) is most associated with HPV 16 and 18 and can manifest as precancerous lesions in the oropharynx and anogenital tract.

In the modern HAART era, the rates of AIDS-defining cancers have plummeted for those who have access to antiviral therapy. In contrast, as survival of PLWH has improved and now rivals that of age-matched controls, rates for HSIL evolving into invasive anal cancer in HIV-positive individuals are increasing. This is despite the obvious and unequivocal benefits of HAART.^27,28^

## PREVALENCE AND INCIDENCE

Risk factors for anal cancer are reviewed in Table 3. Among the groups with the highest prevalence of HPV infection are men who have sex with men (MSM). For those who are also HIV seropositive, the vast majority will have a co-occurring HPV infection. They represent the population with the highest prevalence of HPV infevtion.^27^ It is estimated that high-risk HPV types are present in over 70% of HIV-positive MSM.^29^ HPV-16 is linked to more than one half of cases of HSIL and anal cancer. Because high-risk HPV is more common in MSM who are also HIV seropositive, they will be most vulnerable to anogenital HPV-related malignancies.^29^ Anal HPV is highly prevalent in PLWH, so it follows that anal HSIL is also common in this population.

In the AIDS Cohort Study, investigators noted that abnormal anal cytology was correlated inversely with absolute CD4+ T-lymphocyte cell count. Among HIV-infected MSM, the prevalence of abnormal anal cytology was 38% with a current CD4+ count of >500 cells/mm^3^, 41% with a CD4+ count between 350–499 cells/mm^3^, and 47% with a CD4+ count <350 cells/mm^3^.^30^ A prospective cohort study of anal HPV infection in HIV-infected MSM revealed that the incidence of any anal HPV infection and oncogenic anal HPV infection was 21.3/100 and 13.3/100 person-years, respectively.^31^ Low CD4+ cell count is a risk factor for HSIL in patients with HIV. Silverberg and colleagues reported that among PLWH and who had a CD4+ count less than 200 cells/mm^3^ there was a three-fold increase in progression of LSIL to HSIL.^32^ In Western counties, the age-adjusted rate during the last decades for anal cancer has increased by 2.2% per year, driven largely by HIV-positive MSW and survivors of organ transplants.^33,34^

## RATIONALE FOR SCREENING

The etiologic role of HPV in the pathogenesis of cervical intraepithelial neoplasia (CIN) and invasive cervical cancer is well appreciated. Tools available to the clinician such as pap smears and cervical colposcopy are used for screening and early detection. Precancerous lesions are identified and removed before they become malignant. As is true for anal cancer, HPV is associated with 95% of cervical cancers with oncogenic HPV types 16 and 18 accounting for more than half of cervical malignancies.^35^

For cervical cancer screening, the benefits of “screen and treat” are incontrovertible. Such an approach has led to a dramatic decline in advanced cervical cancer in those countries with robust gynecological and cancer control programs in place. An analogous approach for anal cancer screening whereby precancerous lesions are identified and then treated has been endorsed by the New York State Public Health Department, but the practice has remained controversial and not always covered by insurance plans. High resolution anoscopy (HRA) is considered the gold standard for detection of HSILs.^36^ The first prospective study exploring a surgical intervention for HSIL demonstrated that HRA-guided excision or cauterization was effective in diminishing HSIL in HIV-negative patients.^37^ The various modalities to treat HSIL include targeted destruction under HRA, the application of topical agents (5-Fluorouracil [5-FU], imiquimod, and trichloroacetic acid [TCA]) to lesions, and less commonly wide local excision. The later approach can be quite painful. Consequently, patients who undergo surgical ablations are less likely to consent to further evaluations. With the applications of topical therapies pain is less of an issue and compliance with follow-up and additional treatments may be improved. Yet, regardless of the modality used to treat anal lesions, high rates of HSIL persistence and recurrence occur with ongoing follow-up.^38–40^ Risk factors for recurrence of HSIL include living with HIV and persistent immunosuppression and increasing extent of disease.

In a retrospective evaluation of HIV-infected MSM diagnosed with anal cancer between 1997 to 2011, many of the patients were diagnosed with anal cancer at their first clinic visit, and in each instance, they were also identified as having HSIL.^41^ Yet the risk and progression rates from untreated HSIL to anal cancer among PLWH are not precisely known.^42,43^ In a meta-analysis involving the prevalence and incidence of anal HPV detection, anal intraepithelial neoplasia (AIN) and anal cancer in MSM, the progression rate from anal HSIL to anal cancer among HIV-infected men in the HAART era was 5.1 per 100,00 men.^44^

*The New England Journal of Medicine* published the results of the Anal Cancer/HSIL Outcomes Research (ANCHOR) trial in June 2022.^45^ Because of higher-than-expected progression rates from HSIL to cancer, the final target number of participants progressing to anal cancers was reached before enrollment was completed, and further randomization of study participants to either the treatment or active-monitoring groups was halted after a final analysis by the ANCHOR trial Data and Safety Monitoring Board.

The results of the ANCHOR study will likely lead to significant practice changes for it shows for the first time that through aggressive screening and treating of HSIL, a highly trained HRA proceduralist can mitigate the risk of progression to anal cancer in a high-risk population of PLWH.

## ANCHOR TRIAL DESIGN

The ANCHOR study is a randomized phase III clinical trial which enrolled participants (n = 4446) across 25 cities in the United States.^45^ PLWH who at time of enrollment were 35 years of age or older and who had biopsy-proven HSIL (AIN grade 3 [AIN3] or HPV p16-positive AIN2) were eligible to take part in the study. The primary outcome was progression to anal cancer in a time-to-event analysis. A secondary outcome was to evaluate the safety of treatments for anal HSIL.

The ANCHOR study excluded individuals with a history of preexisting anogenital cancer. The study also initially excluded participants who had received HSIL treatment before study enrollment and those who had received the HPV vaccine, but these requirements were discontinued about mid-way through the study. Participants were randomized 1:1 to an *intervention group* or to *an active monitoring group*. The intervention group received either ablative or topical treatment for HSIL at the clinician’s discretion. Ablative treatments included thermal ablation (hyfrecation/electrocautery), ablation or excision under anesthesia, infrared photocoagulation, laser therapies, and topical imiquimod and topical 5-fluorouracil (5-FU). The trial was not designed to determine which therapy was most effective in ablating HSIL; however, most participants were treated with office-based electrocautery (primarily hyfrecation).

Volunteers from the control group underwent active monitoring of HSIL, including clinical examinations with HRA every 6 months and lesion biopsies every 12 months. At each clinic assessment, anal swabs were also collected for cytological analysis. Participants from both the intervention and active monitoring groups who were identified with lesions which were concerning for rapid progression to cancer could be seen as frequently as every three months. There were no limitations placed on the investigators for when they should collect anal biopsies if or when they had concerns for malignancy. Those individuals who were diagnosed with invasive cancer were taken off the study and were promptly referred for anal cancer-specific evaluation and therapy.

## ANCHOR STUDY RESULTS

**Figure 1.**
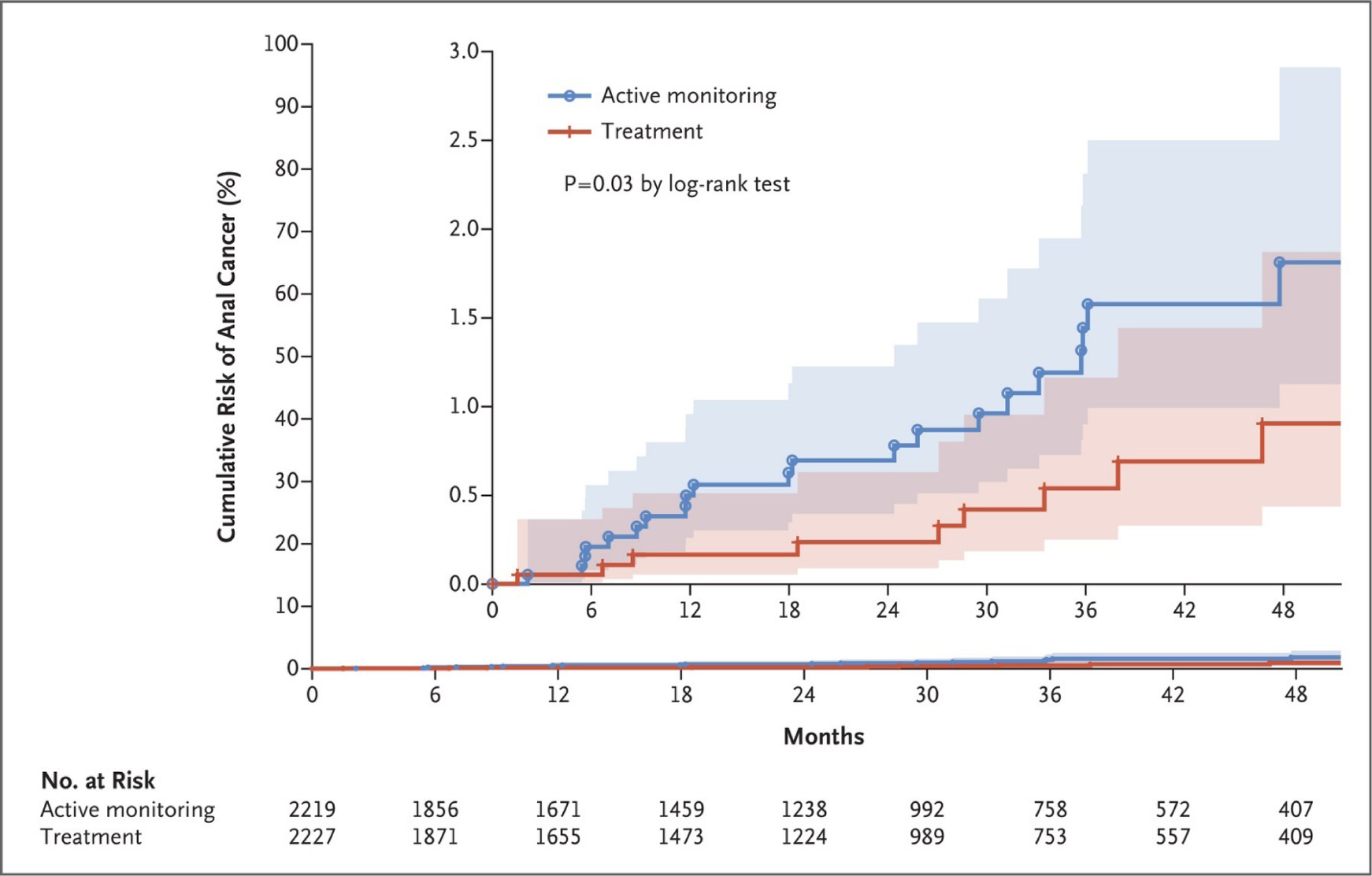
Kaplan–Meier Curve of the Time to Progression to Anal Cancer. The inset shows the data on an expanded y axis. The shaded areas represent 95% confidence intervals. From *The New England Journal of Medicine*, Palefsky JM, Lee JY, Jay N, Goldstone SE, Darragh TM, Dunlevy HA, Rosa-Cunha I, Arons A, Pugliese JC, Vena D, Sparano JA, Wilkin TJ, Bucher G, Stier EA, Tirado Gomez M, Flowers L, Barroso LF, Mitsuyasu RT, Lensing SY, Logan J, Aboulafia DM, Schouten JT, de la Ossa J, Levine R, Korman JD, Hagensee M, Atkinson TM, Einstein MH, Cracchiolo BM, Wiley D, Ellsworth GB, Brickman C, Berry-Lawhorn JM; ANCHOR Investigators Group. Treatment of Anal High-Grade Squamous Intraepithelial Lesions to Prevent Anal Cancer 2022; 386:2273–2282. Copyright © 2022 Massachusetts Medical Society. Reprinted with permission from the Massachusetts Medical Society.

**Figure 2.**
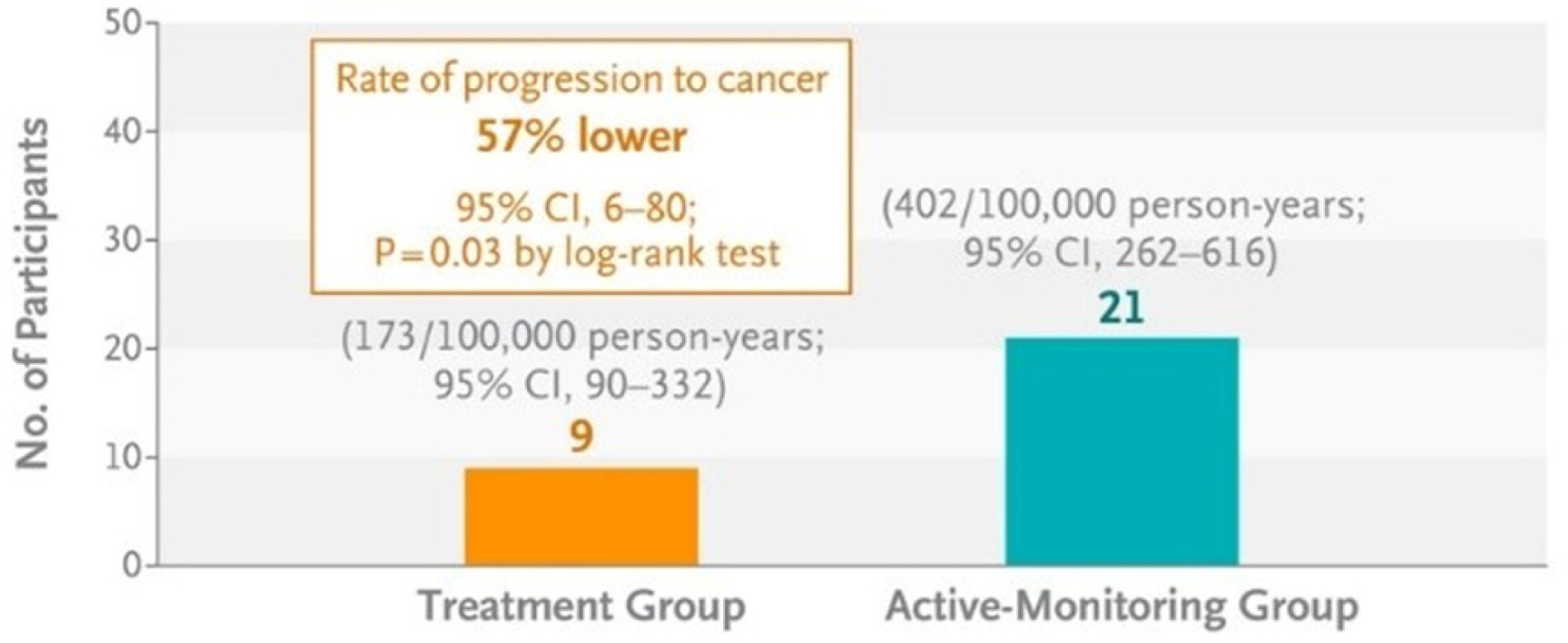
Invasive anal cancer (median follow-up 25.8 months). From *The New England Journal of Medicine*, Palefsky JM, Lee JY, Jay N, Goldstone SE, Darragh TM, Dunlevy HA, Rosa-Cunha I, Arons A, Pugliese JC, Vena D, Sparano JA, Wilkin TJ, Bucher G, Stier EA, Tirado Gomez M, Flowers L, Barroso LF, Mitsuyasu RT, Lensing SY, Logan J, Aboulafia DM, Schouten JT, de la Ossa J, Levine R, Korman JD, Hagensee M, Atkinson TM, Einstein MH, Cracchiolo BM, Wiley D, Ellsworth GB, Brickman C, Berry-Lawhorn JM; ANCHOR Investigators Group. Treatment of Anal High-Grade Squamous Intraepithelial Lesions to Prevent Anal Cancer 2022; 386:2273–2282. Copyright © 2022 Massachusetts Medical Society. Reprinted with permission from the Massachusetts Medical Society.

Nine participants in the treatment group were diagnosed with invasive anal cancer and 21 participants in the active-monitoring group were diagnosed with anal cancer. The observed rate of progression to cancer in the treatment group was 173 per 100,000 person-years (95% confidence interval ([CI], 90 to 332) of follow-up. In the active monitoring group, the rate of progression to cancer was 402 per 100,000 person-years (95% CI, 262 to 616).^45^ This resulted in a 57% (95% CI, 6 to 80; P = 0.03 by log-rank test) reduction in cancer among those in the treatment group compared to those in the active-monitoring group. The cumulative incidence of progression to anal cancer at 48 months was 0.9% in the treatment group and 1.8% in the active-monitoring group (Figures 1–2). The various treatment modalities were associated with a low incidence of serious adverse events.

The rate of progression to cancer among the participants in the active-monitoring group, at 402 per 100,000 person-years, was higher than expected on the basis of published estimates from cancer–HIV registry matches, even after accounting for all the trial participants having HSIL.^46^ The ANCHOR study authors speculate that these findings may be due to early cancer detection; in the absence of screening, anal cancer is usually diagnosed after the development of symptoms such as rectal bleeding and anal pain. They point out that the percentage of stage I or II cancers that were diagnosed in the active-monitoring group was higher than that reported in national data.^47^ This result may also be due to an enriched group of participants who were 35 years of age or older, many of whom were smokers, and were more likely to have sex with men than in the overall U.S. population of PLWH.

In ANCHOR, not all anal cancers were prevented in the treatment group even with aggressive treatment of HSIL lesions. The study authors point to a similar dilemma in cervical and colon cancer screening and treatment where efforts to treat cervical HSIL or colon polyps can still lead to breakthrough cancers, particularly if lesions are large and margins of resection are imperfect.^35,48–52^ Appropriately, the authors stress that reduction in smoking and obtaining HPV vaccines at a young age are important as they can also mitigate the risk of invasive cancer.

## LESSONS LEARNED

Strengths of the ANCHOR trial include that it incorporated a large, multicenter, diverse population of participants with characteristics that mirror those of the overall U.S. population of PLWH.^45^ Trial procedures were performed by clinicians who all had undergone rigorous training in the performance of HRA and who were uniformly subject to pretrial qualification and ongoing quality assurance. Central pathological review was also performed on nearly all biopsy samples with positive results for HSIL during screening and in 100% of cases of anal cancer during the trial.

Also worth noting, the ANCHOR study includes participants who face stigmatization because of their sexual orientation, their ethnicity, and the type of cancer that is being addressed by the study investigators. They face huge challenges in receiving medical care and typically do not enroll or are excluded from participating in cancer control and clinical trials.

Essential to the success of ANCHOR were novel outreach efforts that incorporated a very engaged and empowered community advisory board ‒ many of whom had years of experience representing their constituents on a local and national level. At some participating sites, peer navigation for study volunteers was also available. The availability of nominal compensation for study volunteers to help defray costs of travel, food, and missed work was also likely very important in promoting retention of study volunteers. Having clinicians who are passionate and experienced in working with this often socially- and medically marginalized group of study participants was also an important component of the success of this study.^53^

## FUTURE EFFORTS

Ironically, factors that make ANCHOR unique also may contribute to challenges with generalizing study results more widely. Future efforts will need to address the educational needs to train a new generation of HRA experts and the infrastructure needed to make such screening and treatment more readily available to high-risk groups. Although HRA is well tolerated, it is a cumbersome and time-dependent procedure that requires much cooperation from the patient. The encouraging results from ANCHOR demonstrating that a screen and treat approach can impact anal cancer rates belies the importance of developing non-invasive methods to more easily and better screen high-risk groups. How best to incorporate emerging technologies such as circulating tumor DNA into cancer screening algorithms is also an important question and applies to anal cancer as well.^54^

The study investigators plan to analyze data regarding quality of life of participants who participated in ANCHOR and best practices that lead to improved outreach and retention efforts not just for ANCHOR participants but for the design and implementation of future investigations in this population. The ANCHOR study’s use of social media to educate participants on anal cancer risks and screening and investigators efforts to interact more closely with potential participants through frequent community education and feedback projects may be difficult to quantitate but will also be important areas of investigation.

An important additional area of investigation will come through analyzing all records of those who were diagnosed with anal cancer and taken off the ANCHOR study. Was their survival potentially enhanced by identification of anal cancer at an earlier cancer stage? Also, were similar cancer treatment standards practiced across the various ANCHOR sites in the treatment of these individuals.

The ANCHOR trial findings will be foundational in moving the field of anal cancer prevention forward. Although questions remain, including the impact of such efforts on quality of life, these important study results will provide the basis for better defining clinical guidelines for the screening and treatment of HSIL in PLWH.

Although the study included only PLWH who were 35 years-of-age or older with high grade anal dysplasia, the ANCHOR study results imply potential benefit in other high-risk populations, including HIV-negative MSM, immunocompromised individuals including those who have undergone organ transplants, men with a history of penile cancer and women with a history of cervical and vulvar cancers.

The ANCHOR study has been widely hailed as providing strong evidence that strategies aimed at preventing anal cancer are possible. It has, however, been pointed out that monitoring by HRA is not readily adoptable on a global scale owing to the paucity of certified anoscopists and the impact of health care costs.^55^ Recognizing the relatively low yield and high burden of frequent HRA assessment on patient quality of life will also need to be studied to better refine surveillance-interval timing.^56^

The development of robust biomarkers for anal cancer whether collected from anal swabs, menstrual pad samples, plasma-based circulating tumor DNA (ctDNA), and the analysis of ctDNA obtained from non-blood bodily fluids offer potential benefits with less cumbersome methods of screening for anal cancer than current cytology and HRA-based efforts.^57,58^ However, well-designed clinical trials in which the results are used to inform treatment decisions and that demonstrate meaningful benefits to patients will be necessary before such assays are to be broadly implemented clinically.

## Figures and Tables

**Table 1.** HIV-Related Tumors and Their Associated Viruses

AIDS-Defining Malignancies	Virus

Kaposi sarcoma	HHV-8
Intermediate and high-grade NHL	EBV and rarely HHV-8
Primary effusion lymphoma	HHV-8/EBV (rarely)
PCNSL	HPV
Invasive Cervical Cancer	HPV

**Non-AIDS-Defining Malignancies**	

Hodgkin’s lymphoma	EBV
Low Grade B Cell NHL	HCV (rarely)
Non-cervical anogenital Cancers	HPV
Nasopharyngeal carcinoma	EBV
Conjunctival Squamous cell cancer	HPV
Spindle cell tumors	EBV
Liver Cancer	HBV and HCV
Papillomatosis-neoplastic dermatoses	HPV
Adult T-cell leukemia/lymphoma	HTLV-I
Hepatoma	HBV and HCV
Lung cancer	HPV (rarely)
Merkel cell	MCP
Nonmelanoma skin cancer	MCP

HHV-8 = human herpesvirus Type 8; EBV = Epstein Barr Virus; HPV = human papillomavirus; HBV = Hepatitis B Virus; HCV = Hepatitis C Virus; HTLV-I = Human T-Cell Leukemia Virus Type- I; MCP= Merkel Cell Polyomavirus

**Table 2.** Number of HPV-Associated and Estimated Number of HPV-Attributable Cancer Cases per Year in the United States

Cancer site	Average number of cancers per year in sites where HPV is often found (HPV-associated cancers)	Percentage probably caused by any HPV type^a^	Estimated number probably caused by any HPV type^a^

Cervix	12,293	91%	11,100
Vagina	879	75%	700
Vulva	4,282	69%	2,900
Penis	1,375	63%	900
Anus^b^	7,531	91%	6,900
Female	5,106	93%	4,700
Male	2,425	89%	2,200
Oropharynx	20,839	70%	14,800
Female	3,617	63%	2,300
Male	17,222	72%	12,500
**TOTAL**	47,199	79%	37,300
Female	26,177	83%	21,700
Male	21,022	74%	15,600

a HPV types detected in genotyping study; most were high-risk HPV types known to cause cancer (Saraiya M, et al. U.S. assessment of HPV types in cancers: implications for current and 9-valent HPV vaccines. *Journal of the National Cancer Institute* 2016;107: djv086. Estimates were rounded to the nearest 100. Estimated counts might not sum to total because of rounding.

b Includes anal and rectal squamous cell carcinomas.

Data are from population-based cancer registries participating in CDC’s National Program of Cancer Registries (NPCR) and/or the National Cancer Institute’s Surveillance, Epidemiology, and End Results (SEER) Program for 2015 to 2019, covering 99% of the U.S. population.

Table is reproduced from: U.S. Centers for Disease Control and Prevention. How Many Cancers Are Linked with HPV Each Year? Last Reviewed: October 3, 2022. https://www.cdc.gov/cancer/hpv/statistics/cases.htm#print **Data source:** National Program of Cancer Registries SEER*Stat Database: U.S. Cancer Statistics Incidence Analytic file 1998–2018. United States Department of Health and Human Services, Centers for Disease Control and Prevention. Released June 2021, based on the 2020 submission.

**Table 3.** Risk Factors for Anal Cancer

Anal Cancer Risk Factors	Comments

Chronic exposure to oncogenic HPV types	HPV-16 > HPV-18 to cause anal cancer
HIV infection	Higher rates of smoking and HPV infection
Sexual activity	Multiple sex partners and receptive anal sex
Smoking	Risk may be mitigated by quitting smoking
Lowered immunity	Highest among those with HIV and solid organ transplant recipients
Anogenital cancers other than anal cancer	Penile, vaginal, and vulvar cancers
Gender and race/ethnicity	Anal cancer more common in white women and black men

## Take home message for readers:

- The rates of AIDS-defining cancers are falling for people living with HIV (PLWH) and who have access to active antiretroviral therapies. In contrast, as survival of PLWH has improved and now rivals that of age-matched controls, rates of non-AIDS-defining cancers are increasing. Exposure to oncogenic viruses including human papillomavirus (HPV) as well as to traditional carcinogens, such as tobacco and alcohol are among the reasons for many of these cancers.
- Worldwide, anal cancer rates are increasing, and this is particularly true for high-grade squamous intraepithelial lesions (HSIL) evolving into invasive anal cancer in PLWH.
- The Anal Cancer/HSIL Outcomes Research (ANCHOR) study is a randomized phase III clinical trial which enrolled nearly 4500 participants across 25 diverse cities in the United States. PLWH who at time of enrollment were 35 years of age or older and who had biopsy-proven HSIL were eligible to take part in the study.
- The study is the first to show that screening and treating HSIL in a group at high risk for anal cancer can lead to a reduction in anal cancer incidence.
- The results may not be generalizable to settings in which high-resolution anoscopy screening and treatment are performed by clinicians with less training and support. Additional work is needed to refine screening algorithms for identifying anal HSIL.
